# The Effects of COVID-19 on the Mental Health of Children and Adolescents: A Review

**DOI:** 10.7759/cureus.56473

**Published:** 2024-03-19

**Authors:** Anwar A Sayed, Ahmed A El-Gendy, Abdullah K Aljohani, Rudaynah A Haddad, Odai H Taher, Abdulelah M Senan, Abdulmajeed M Qashqari, Basel A Alqelaiti

**Affiliations:** 1 Department of Surgery and Cancer, Imperial College London, London, GBR; 2 College of Medicine, Taibah University, Medina, SAU

**Keywords:** sars-cov-2, underage, adolescence, mental health, covid-19

## Abstract

The COVID-19 pandemic, caused by the SARS-CoV-2 virus, has profoundly affected global health and well-being. As part of the *Coronaviridae* family, SARS-CoV-2 joins a diverse group of viruses found in both humans and various animal species, including bats, camels, and cats. The pandemic has led to widespread social isolation, reduced physical activity, and significant lifestyle changes, posing potential risks to individuals' mental and emotional health. This review aims to explore the implications of COVID-19 on the mental health of children and adolescents, given the limited attention this population has received in the medical literature. Multiple research studies in several countries have found that the COVID-19 pandemic is associated with greater stress levels, depression, anxiety, insomnia, drug misuse, and other mental health challenges among young individuals. Understanding the long-term effects of the pandemic on mental health is crucial for developing effective interventions and support systems to promote resilience and well-being in children and adolescents. Even after the pandemic ends, it is crucial to prioritize understanding the long-term impacts of the pandemic on mental health, integrating findings into public health strategies, addressing mental healthcare disparities, and fostering resilience in children and adolescents. Achieving these objectives requires collaborative efforts across various sectors to ensure equitable access to mental health resources and the implementation of sustainable solutions for the well-being of young people in the aftermath of the pandemic.

## Introduction and background

Coronavirus (CoV) belongs to the *Nidovirales* order, which is a large family of positive-sense single-stranded RNA viruses. This order includes the *Roniviridae*, *Arteriviridae*, and *Coronaviridae* families. The *Coronaviridae* family has two subfamilies: *Torovirinae* and *Coronavirinae*. The *Coronavirinae* subfamily is further subdivided into alpha, beta, gamma, and delta COVs. These categories are based on phylogenetic grouping, which reflects the evolutionary connections among the viral subtypes. Coronaviruses have viral RNA genomes that range in length from 26 to 32 kilobases. These viruses have been found in diverse animal species, such as birds, cattle, and mammals, which encompass camels, bats, masked palm civets, mice, dogs, and cats. Coronaviruses are considered major diseases due to their widespread distribution and infectiousness [1]. They have been linked to illnesses of the respiratory, hepatic, neurological, and gastrointestinal systems in humans [2]. The Middle East respiratory syndrome coronavirus (MERS-CoV), severe acute respiratory syndrome coronavirus (SARS-CoV), and the coronavirus disease 2019 (COVID-19) virus are all examples of this [3]. The COVID-19 pandemic was caused by the severe acute respiratory syndrome coronavirus 2 (SARS-CoV-2), a beta-COV [1]. The first instances were most likely connected to direct contact with infected animals (animal-to-human transmission) at a seafood market in Wuhan, China [4]. Common symptoms of the disease include anosmia, arthralgia, asthenia, chest pain, cough, cutaneous rash, diarrhea, dyspnea, expectoration, fever, lightheadedness, myalgia, odynophagia, rhinorrhea, syncope, vomiting, and weakness. After an incubation period of two to 14 days, most patients typically present these general symptoms, which can persist for one to three weeks [5]. Patients with serious illnesses come with severe pneumonia, and other possible conditions include acute respiratory distress syndrome (ARDS), sepsis, or septic shock [4]. Many stayed home and engaged in fewer social interactions and physical activities due to the COVID-19 pandemic as part of the imposed restrictions [6,7]. These measures can be harmful to both physical and mental health. Working from home, being unemployed, homeschooling children, and being out of touch with other family members, friends, and coworkers are all new realities that take time to adjust to. It was tough for all to adjust to such lifestyle changes, let alone deal with the fear of contracting the virus and worry for those close to us who are more vulnerable. These changes can be particularly challenging for those who suffer from mental problems [8]. During the COVID-19 pandemic, Carter et al. did a comprehensive review to synthesize current research on the prevalence of depression, anxiety, post-traumatic stress disorder (PTSD), and other types of psychological distress in the general population. During the COVID-19 pandemic, there was a notable surge in rates of anxiety (from 6.33% to 50.9%), depression (from 14.6% to 48.3%), post-traumatic stress disorder (from 7% to 53.8%), psychological distress (from 34.43% to 38%), and stress (from 8.1% to 81.9%) within the general population in China, Spain, Italy, Iran, the United States, Turkey, Nepal, and Denmark [9]. Al Dhaheri et al.'s research discovered that between May and June 2020, 6142 people from 18 Middle East and North Africa (MENA) nations completed an online questionnaire to assess the psychological impact of COVID-19. COVID-19 made most people feel afraid, anxious, or helpless (45-62%). Furthermore, more than 40% reported that job and financial worries were causing them more significant stress. The Impact of Event Scale-Revised (IES-R) scores were higher for females, those between the ages of 26 and 35 years, those with a lower educational level, and those from North Africa. Since the pandemic, more than 42% say they have received more help from family members, 40.5% say they have paid more care to their mental health, and more than 40% say they have spent more time resting [7]. With a high part of the population falling between the ages of 10 and 20 years, minor persons make up a large share of the patient demographics in our community. That means they make a significant contribution to the community [10]. Despite its relevance in terms of the community's general mental health problems, the influence of COVID-19 on the mental health of minors has not been thoroughly explored or published in the medical community. As a result, this narrative review aims to understand more about COVID-19's impact on our children's mental health.

## Review

Adolescent mental health

Mental health may be described as the absence of mental illness or as a condition influenced by biological, psychological, and social elements that affect one's mental state and ability to function in society [9,10]. Other connotations include intellectual, emotional, and spiritual growth, positive self-perception, emotions of self-worth, physical health, and intrapersonal harmony. Prevention techniques try to reduce the occurrence of mental illness, whereas promotion tactics enhance mental wellness [10]. The World Health Organization (WHO) defines mental health as "a condition of wellbeing in which the individual recognizes his or her potential, can cope with the usual demands of life, can work successfully and fruitfully, and contribute to his or her community" [11].

Manwell et al. involved 50 mental health experts from eight countries who completed an online survey to assess the adequacy of current definitions of mental health and identify core concepts. A qualitative thematic analysis was conducted on their responses. Participants' core concepts of mental health were primarily focused on personal issues, such as competence and the capacity to choose how to interact with society. The notions of agency, autonomy, and control were frequently highlighted throughout the responses, especially concerning the individual's capacity or aptitude to cope with and change their environment. Respondents also identified the self as an essential component of mental health, demonstrating that an individual's subjective experience is critical to well-being, mainly when accomplishing one's goals [10].

Because of their impact on so many elements of daily life, basic cognitive and social abilities are seen as crucial components of mental health. Social skills are the ability to interact and connect with others through one's verbal and nonverbal abilities. The capacity to pay attention, retain and organize information, solve problems, and make decisions are all examples of cognitive talents. These skills come together to allow humans to function in their surroundings. The concept of the "basic" level of these skills is intended to emphasize that minor impairments can still be consistent with mental well-being. However, if impairments are more significant and not balanced by other factors, individuals may need support from society, including assistance in finding employment, financial aid, or ad hoc training programs [12]. Many mental health disorders start in adolescence and contribute to the present disease burden in youth and later life. More than half of all adult mental health issues start before 18 years. Adolescent pregnancy, human immunodeficiency virus (HIV)/acquired immunodeficiency syndrome (AIDS), other sexually transmitted illnesses, marital violence, child abuse, vehicle accidents, physical confrontations, crime, homicide, and suicide have all been connected to poor mental health. Neuropsychiatric illnesses are the leading cause of years lost due to disability in those aged 10 to 24 years, accounting for 45% of all years lost owing to impairments globally [13]. Adolescents are in a sensitive developmental stage, with the majority of mental problems manifesting at this time [14]. According to the diagnostic criteria of mental health disorders, about one in every seven youngsters has a mental health problem. Untreated mental health difficulties in children and adolescents are associated with poor health, academic, and social outcomes, as well as greater rates of drug misuse, self-harm, and suicide, and they frequently persist into adulthood. Indeed, half of all lifetime mental health difficulties begin before the age of 15 years and three-quarters before 18 years, resulting in a significant global socioeconomic cost [15,16]. These unfavorable short- and long-term repercussions of adolescent mental health issues highlight the importance of early detection and expert treatment. Teenage mental health issues can be treated with effective, evidence-based therapies. On the other hand, only around two-thirds of young people with mental health concerns and their families seek professional care [15]. Rickwood et al. looked at the major roadblocks and facilitators young people face while seeking help for mental health issues. The major roadblocks they observed were a lack of emotional competency, unfavorable attitudes toward seeking help, and stigma. On the other hand, the key facilitators were emotional competence, past good contacts with health practitioners, and mental health literacy [17,18].

COVID-19 pandemic and mental health

The COVID-19 pandemic has had far-reaching effects on people's lives. The pandemic's influence on nearly every sector of society has been well documented, including the economy, education, public health, and health care. In several nations, psychologists and mental health professionals have raised the fear that the pandemic will worsen mental health difficulties [19]. Fear and anxiety about contracting the virus, uncertainty about testing and medical care, stress from social isolation and lockdowns, job loss and rising medical expenditures, and the social stigma of being sick all have the potential to impact mental health negatively. Deteriorating mental health and social stress may aggravate suicidal ideation and, in the worst-case scenario, suicide mortality [20]. Multiple studies in many nations have indicated that the COVID-19 pandemic is linked to higher stress levels, anxiety, depression, insomnia, and drug abuse [19]. In affluent nations, the majority of research on the pandemic's relation to mental health and suicidal behavior has taken place [21].

Nearly four out of 10 people in the United States have had anxiety or depression throughout the pandemic [22]. According to a July 2020 Kaiser Family Foundation (KFF) Health Tracking Poll, many adults are reporting specific adverse effects on their mental health and well-being as a result of their concern and stress about the coronavirus, such as difficulty sleeping (36%), eating (32%), increasing alcohol consumption or substance use (12%), and worsening chronic conditions (12%). As the pandemic spreads, ongoing and mandatory public health measures expose more people to situations linked to poor mental health outcomes, such as isolation and job loss [23].

The effects of the pandemic on the mental health of children and adolescents

Across the research evaluated, the frequency of mental health concerns during the COVID-19 pandemic differed by age group. The data reported here are divided into three categories: younger children, school-age children, and adolescents. For younger children (typically under seven years old), research shows an increase in reports of clinginess, fear of safety, uncooperative and worry behaviors, as well as boredom, attention-seeking, and anxiety, particularly among children of hourly service workers experiencing significant stressors related to COVID-19 [24].

School-aged children (ages seven to 13 years) had greater rates of anxiety and depression than before the pandemic. Studies have shown substantial depression symptoms ranging from 2.2% to 11.78%, as well as significant anxiety symptoms ranging from 1.8% to 23.87%. These children have demonstrated increased inattention, a need for reassurance, academic problems, inappropriate behavior, anxiety, social isolation, and depression [24].

Parents have expressed worry about sadness, anxiety, misbehavior, social isolation, attention challenges, and impulsivity in their adolescents. Self-reported rates of substantial anxiety symptoms ranged from 10.4% to 29.27%, while rates of severe depressive symptoms ranged from 17.3% to 22.28%, with female adolescents having a greater frequency. High school seniors had the greatest rates of depression, anxiety, and stress. During the post-pandemic months, mental health-related emergency department visits increased among adolescents aged 12 to 17 years, with females making up the majority. A considerable proportion of college-aged youngsters reported symptoms of internalizing illnesses, such as sadness or anxiety, while others reported externalizing disorders, such as violence or oppositional behavior. Somatic symptoms were also common, especially among individuals concerned about satisfying daily needs [24].

Substance abuse is a concern among adolescents as well. Prior to the pandemic, 15% of high school students had engaged in illicit drug use, while 14% had misused physician-prescribed opioids. Solitary drug use (as opposed to social use) has increased among youths during the pandemic, linked to worse mental health [25]. Suicidal thoughts are another primary concern for adolescents during the pandemic. Suicide is the second largest cause of mortality among those aged five to 24 years in the United States, and it is a major public health problem. According to data, depression, anxiety, and social isolation increased during the COVID-19 pandemic, potentially contributing to a higher risk of suicide among adolescents. During the COVID-19 pandemic, suicide rates surged by 31% more than expected. Suicide rates among adolescents in the United States increased by 135% between 2010 and 2019. Recognizing child suicide as a major public health concern, the National Institute of Mental Health convened a research discussion series in 2021 to explore viable ways to reduce child suicide risk by addressing critical knowledge and research gaps [26].

Educators have a crucial role in discovering and reporting child maltreatment. Many incidents are likely to go undiscovered due to school closures and stay-at-home orders, and at-risk youngsters are more likely to be exposed to their abusers at home [27]. According to Halldorsdottir et al.'s research, the incidence of depression symptoms grew from 2016 to 2018, then steadily increased from 2018 to levels reported during the COVID-19 pandemic in 2020. Some adolescent groups are more likely than others to experience mental health problems. Girls were more likely than males to report the negative effects of the COVID-19 pandemic on a variety of life dimensions, including academic achievement, peer relationships, and mental health. During the pandemic, boys were more likely to have a positive attitude toward their daily lives. Furthermore, girls were more concerned about particular pandemic-related variables influencing mental health, such as fear of contracting COVID-19 and changes in daily routines. Overall, these data indicate that females may be more susceptible to mental health difficulties during the pandemic [28].

Girls reported more regions badly impacted by the COVID-19 pandemic than boys did. Significant gender disparities occurred in terms of which COVID-19 pandemic-related characteristics the adolescents saw as having a negative impact on their mental health. Specifically, compared to adolescent boys, girls were significantly more likely to consider worrying about themselves, others close to them contracting COVID-19, spending more time at home, not being able to see friends in person, worrying about people becoming infected around the world, a change in school routine, more time with family, increased stress due to changes in daily routines, and media coverage of the pandemic. On the other hand, females assessed more characteristics related to the COVID-19 pandemic as having a favorable impact on their mental health than boys. Gender inequalities occurred in terms of which components of the COVID-19 pandemic benefited teenage mental health. They were more likely to report that spending more time with family, using social media, watching TV, and sleeping, as well as having more flexibility in their daily routine, improved their mental health [28]. According to analogous research undertaken in Norway, particular categories of youth, such as those living in divided families, are disproportionately at risk for heightened symptoms of mental health concerns during times of crisis, such as the COVID-19 pandemic. Monitoring mental health trajectories in girls and boys of various ages, as well as in diverse teenage communities, will allow for better-focused interventions to minimize psychological disease [29].

The importance of social support in deciding teens' mental health during times of crisis, such as the pandemic, was also demonstrated in this study. Adolescents reported high levels of low to moderate social support throughout the pandemic, which led to increased anxiety and despair [30].

Octavius et al. conducted a systematic review on the influence of COVID-19 and related factors, such as lockdown measures on adolescent mental health. Their analysis revealed a correlation between COVID-19 and alterations in adolescent mental health, underlining the necessity for mental health considerations in COVID-19 management strategies [31].

Rosen et al. did a study integrating two longitudinal samples of children and adolescents to explore pandemic-related stresses, internalizing and externalizing psychopathology, and putative protective variables. Rosen et al. demonstrated that both internalizing and externalizing psychopathology rose significantly throughout the pandemic. Higher levels of exposure to pandemic-related stressors were linked to an increase in internalizing and externalizing symptoms early in the pandemic and six months later. Having a structured schedule, less passive screen time, less exposure to pandemic news media, and, to a lesser extent, spending more time in nature and getting enough sleep were all related to decreased psychopathology. The relationship between pandemic-related stressors and psychopathology was reduced in youths with limited passive screen time and was missing in children, but not adolescents, with lower pandemic-related news media intake [32]. The majority of advice focuses on helping children and adolescents who exhibit stress or anxiety, helping them find healthy outlets for their emotions, like play or drawing, advice on sticking to routines, advice on keeping kids in touch with caregivers in the event that they are separated from them, and advice on staying in social contact with peers [33].

It is imperative to reflect on the lasting implications for children and adolescents' mental health. Despite the end of the pandemic, the psychological repercussions may persist, emphasizing the need for ongoing assistance and intervention for young individuals who have endured heightened stress, anxiety, and social isolation. Moving forward, the focus in the field should center on comprehending the prolonged effects of the pandemic, incorporating newfound knowledge into public health strategies, tackling mental health disparities, and nurturing resilience among children and adolescents. This necessitates a collaborative effort involving policymakers, healthcare professionals, educators, families, and communities to ensure fair access to mental health assistance and the formulation of sustainable solutions for the well-being of children and adolescents in a post-pandemic world.

## Conclusions

The COVID-19 pandemic has brought to the forefront the critical importance of addressing children and adolescents' mental health amidst global crises. As we move beyond the immediate impacts of the pandemic, it is clear that the psychological toll on children and adolescents remains a pressing concern. Recognizing the long-term implications of heightened stress, anxiety, and social isolation, it is imperative that research and public health efforts prioritize understanding and addressing these challenges. By investing in comprehensive support systems, integrating innovative interventions, and fostering resilience within children and adolescent populations, we can strive toward building a more mentally resilient and healthy generation. Collaboration across sectors and communities will be essential in ensuring equitable access to resources and creating sustainable solutions to support the well-being of adolescents in the post-pandemic era.
